# Topographic characterisation of dental implants for commercial use

**DOI:** 10.4317/medoral.20333

**Published:** 2016-07-31

**Authors:** Amparo Mendoza-Arnau, Manuel-Francisco Vallecillo-Capilla, Miguel-Ángel Cabrerizo-Vílchez, Juan-Ignacio Rosales-Leal

**Affiliations:** 1 Stomatology Department, Dental Prosthesis. Faculty of Dentistry, University of Granada, Spain; 2 Stomatology Department, Buccal Surgery. Faculty of Dentistry, University of Granada, Spain; 3 Applied Physics Department, Bicolloids and Fluids Physics. Faculty of Science, University of Granada, Spain

## Abstract

**Background:**

To characterize the surface topography of several dental implants for commercial use.

**Material and Methods:**

Dental implants analyzed were Certain (Biomet 3i), Tissue Level (Straumann), Interna (BTI), MG-InHex (MozoGrau), SPI (Alphabio) and Hikelt (Bioner). Surface topography was ascertained using a confocal microscope with white light. Roughness parameters obtained were: Ra, Rq, Rv, Rp, Rt, Rsk and Rku. The results were analysed using single-factor ANOVA and Student-Neuman-Keuls(*p*<0.05) tests.

**Results:**

Certain and Hikelt obtained the highest Ra and Rq scores, followed by Tissue Level. Interna and SPI obtained lower scores, and MG-InHex obtained the lowest score. Rv scores followed the same trend. Certain obtained the highest Rp score, followed by SPI and Hikelt, then Interna and Tissue Level. MG-InHex obtained the lowest scores. Certain obtained the highest Rt score, followed by Interna and Hikelt, then SPI and Tissue Level. The lowest scores were for MG-InHex. Rsk was negative (punctured surface) in the MG-InHex, SPI and Tissue Level systems, and positive (pointed surface) in the other systems. Rku was higher than 3 (Leptokurtic) in Tissue Level, Interna, MG-InHex and SPI, and lower than 3 (Platykurtic) in Certain and Hikelt.

**Conclusions:**

The type of implant determines surface topography, and there are differences in the roughness parameters of the various makes of implants for clinical use.

**Key words:**Implants for clinical use, topography, confocal microscopy.

## Introduction

Dental implants are becoming more widespread in daily practice. There are numerous manufacturers of implants that offer greatly varying surface treatments (1,2).

Surface treatment is vital for quick and lasting osseointegration (3-5). Depending on the treatment applied, the topography will differ and the roughness parameters will vary (6,7).

The study of surface topography is of paramount importance as it determines cell response (7-9). By optimizing the topography, we can use implants in cases where the bone is not very favorable, where the maxillary sinus is elevated, and after performing dental extractions. As a result, the chances of long-term success of implant-supported tooth restorations are greatly improved.

There are several types of surface treatment. To this date, there is no proven surface treatment for clinical use which provides superior surface properties in terms of better adhesion and cell proliferation, which should be standardized for all surfaces.

There are several studies which analyse the topography of implants for commercial use, but topography is described in different ways and the studies use diverse methodologies (10). Measuring and assessment techniques should be standardized.

Thus, it is important to characterise the surface topography of implants for use in patients in a systematic manner, including height, special and hybrid parameters. This type of data will help us better understand the biological response and clinical results, and will improve the success rate of implants. The main aim of this paper is to characterise the surface topography of implants for commercial use.

## Material and Methods

This study analysed implants made by six different commercial manufacturers which use various method of surface treatment.  Table 1 shows these methods. A topographic analysis of each surface was carried out using a confocal microscope with white light (Nikon L150 SENSOFAR, Sensofar, Terrasa, Spain).

**Table 1:**
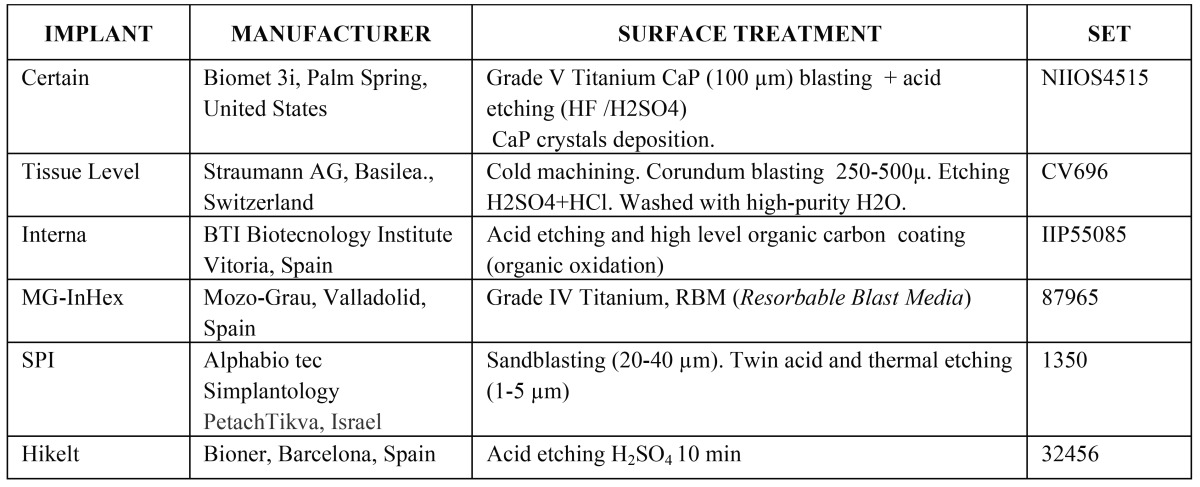
Implant, manufacturer, surface treatment and set of each of the systems analyzed.

 Table 2 shows the topographic parameters analysed (11). Amplitude, average surface roughness (Ra), peak (Rp, relative maximum peak height) and depth (Rv, maximum valley depth) parameters typically offer an approximate description as they only show the topography amplitude without giving any information as to its spatial distribution. However, root mean squared (Rq), skewness (Rsk) and kurtosis (Rku) are statistical moments of peak distribution which describe width, symmetry and shape respectively.

**Table 2:**
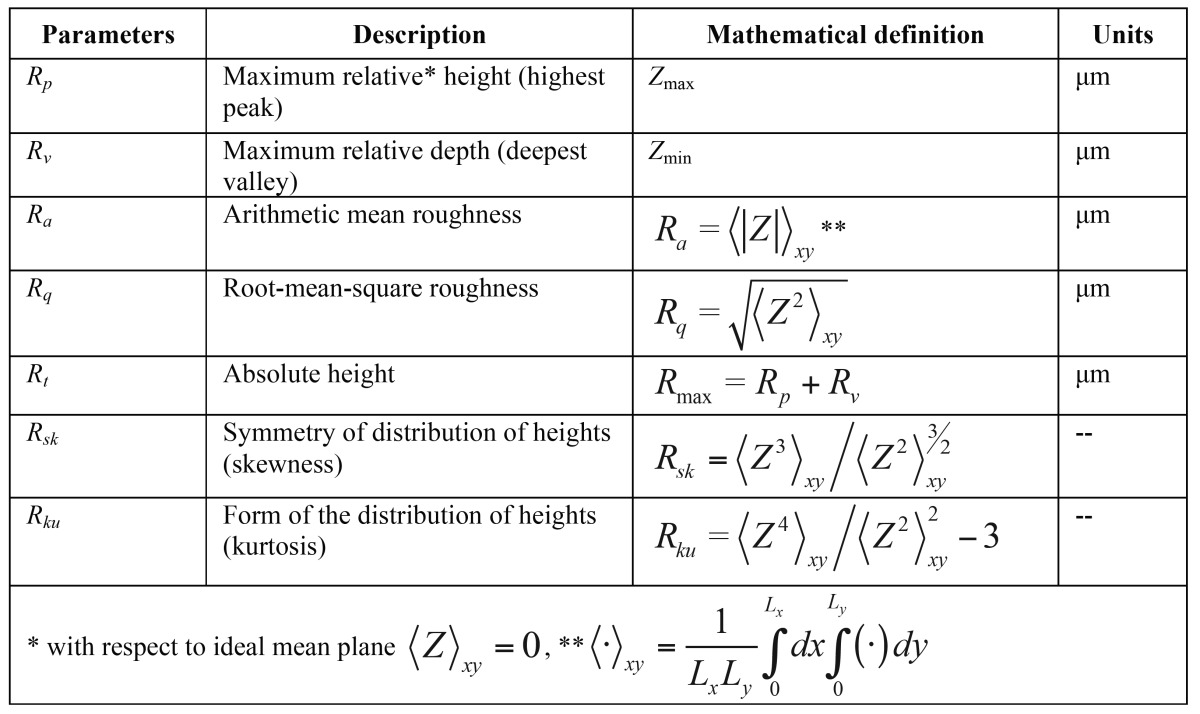
Roughness parameters.

In order to carry out this study, each implant was placed on the stage of a confocal microscope with white light. The implants were taken directly from their blister packaging, ready for clinical use, that is to say that the surface each commercial manufacturer applied to each of the implants had not been altered or modified.

Scans were performed in 292x214 µm2 areas. Five measurements were taken in total for each implant, and three of each of the implants systems were measured. A computer-generated three-dimensional reconstruction of the surface was then used to obtain the roughness parameters to be analysed.

A single-factor ANOVA was carried out in order to analyse the effect that the type of surface of each implant had on surface topography (test factor: implant system; dependent variables: Ra, Rq, Rv, Rp, Rt, Rsk, Rku) (*p*<0.05).

Once ANOVA was carried out, the data obtained was subjected to a Student-Newman-Keuls multiple comparisons test in order to establish the differences between the six types of implant systems (*p*<0.05).

## Results

**Figure 1:**
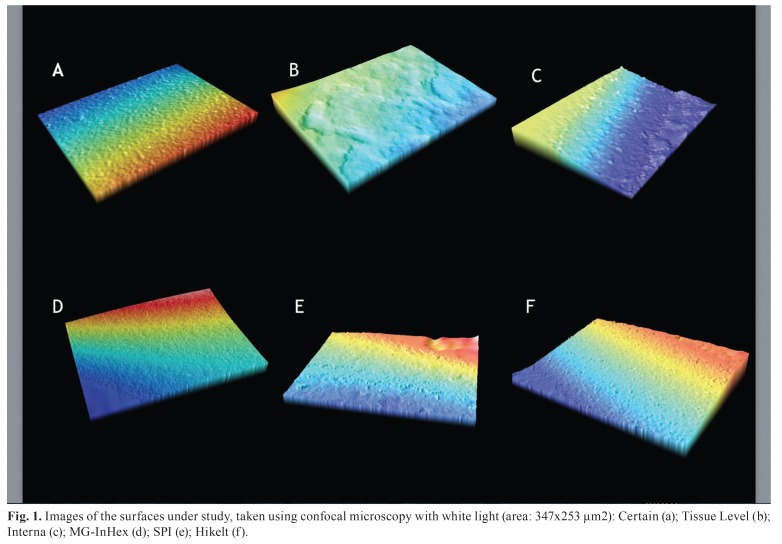
Images of the surfaces under study, taken using confocal microscopy with white light (area: 347x253 μm2): Certain (a); Tissue Level (b); Interna (c); MG-InHex (d); SPI (e); Hikelt (f).

 Table 3 shows the results for the topographic parameters analysed (Fig. 1). shows images of the surface topography of the six implant systems under study, taken using confocal microscopy with white light.

**Table 3:**
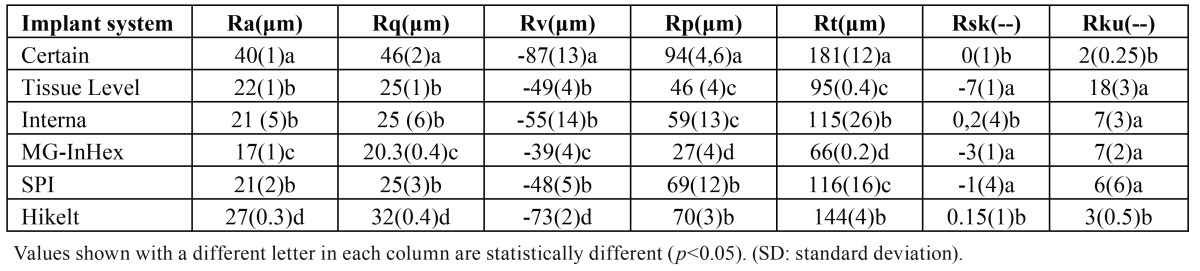
Topographic parameters [mean (SD)].

ANOVA established that the surface of each type of implant had a considerable impact (*p*<0.05) on the topographic parameters analyzed.

The system which obtained the highest Ra scores was Certain, then Hikelt; Tissue Level, Interna and SPI obtained lower scores, and MG-InHex obtained the lowest Ra scores. Rq and Rv scores followed the same trend.

Certain, followed by SPI and Hikelt, obtained the highest Rp (maximum peak height) scores. Interna and Tissue Level obtained lower scores for maximum peak height. MG-InHex obtained the lowest Rp scores.

Rt analysis established that Certain scored the highest, followed by Interna and Hikelt. SPI and Tissue Level had similar lower scores. Mg-InHex was the lowest scoring, there being no statistical differences among the systems analyzed.

Skewness (Rsk) was positive in Certain, Interna and Hikelt surfaces; this means that their surfaces have high peaks and shallow valleys, or pointed surfaces. In MG-InHex, SPI and Tissue Level surfaces Rsk was negative, which means that they have more pores than peaks, that is, they were perforated surfaces.

Kurtosis (Rku) analysis showed four leptokurtic (Kurtosis>3) surfaces: Tissue Level, Interna, MG-InHex and SPI; and two platykurtic (Kurtosis<3) surfaces: Certain and Hikelt. In Leptokurtic surfaces the peaks and valleys are closer together, whereas in platykurtic surfaces the peaks and valleys are less concentrated.

Figure 1 shows images of the various surfaces under study, taken using confocal microscopy. Surface morphology for Certain was pointed, and the peaks showed lower frequency. Surface was homogenous (Fig. 1a). The surface of Tissue Level presented smooth and frequent peaks and valleys, and was abraded (Fig. 1b). The surface of Interna was pointed and continuous, and its appearance was regular (Fig.1c). MG-InHex presented just perceptible pores and peaks, the frequency of the pores being constant (Fig. 1d). SPI presented a continuous surface with few indents, and high peaks in proportion to the valleys (Fig. 1e). Hikelt presented pronounced peaks that were set apart. It was not a smooth and homogenous surface (Fig. 1f).

## Discussion

This study has established topographic differences depending on the type of implant. Topography and, in particular, roughness, are determining factors in the initial stages of osseointegration (12). Topography determines processes such as protein adsorption and osteoblast adhesion (13). It has been proven that roughness promotes gene expression, activates integrins and increases the activity of alkaline phosphatase (14).

The highest surface roughness Ra and Rq scores were found in Certain. The surface treatment used in this system was a type of calcium phosphate (CaP) blasting called DCD (Discrete Crystalline Deposition) and acid treatment (HF/H2SO4). This type of treatment produced the highest roughness. As it has been shown in other papers, blasting increases roughness, and blasting together with etching increases it even more (8,15). Particle size directly determines roughness amplitude (16). In Certain, the CaP particle size used for the blasting was 20-100 µm (17). Compared to all the other systems where blasting was also used (Titanium Dioxide, Aluminium Oxide or Corundum), in this system a variable particle size was used, which explains Rt, Rv and Rp data. Sudden peaks and valleys appeared on a relatively homogenous surface, making the surface more heterogeneous (18) (Fig. 1a).

Hikelt presented lower roughness than Certain, but higher than all the other systems. This system uses an acid treatment (H2SO4). In other studies, acid etching produced less roughness than surface abrasion by blasting (8,15,19). The machining in this system resulted in an initial roughness that was maintained after etching, and this is the reason why it obtained higher Ra and Rq scores than other systems which used particle blasting before acid etching. Etching time and acid concentration also determined roughness amplitude: the longer the time or concentration, the higher the roughness (20). This might explain the Ra and Rq scores obtained by this system (Fig. 1f).

Interna, Tissue Level and SPI obtained similar Ra scores, lower than Hikelt and Certain. Interna used acid etching and high level organic carbon coating (organic oxidation). Its surface presented numerous steep peaks due to high magnification etching, but low height in amplitude deviation; the surface was smooth in microscale and nanoscale (18). This material was not as hard as aluminium oxide or diamond, titanium or hydroxylapatite (21) (Fig. 1c). Tissue Level had Ra and Rq scores similar to Interna. However, Rp, Rv and Rt scores were much higher in Interna. This means that the surface was relatively homogenous, interrupted by occasional pronounced peaks and valleys (Fig. 1c). This is due to abrasion particles not having the same size; particles of bigger than average size produce pronounced peaks and valleys.

Tissue Level used cold machining, followed by 250-500µm corundum blasting (Al2O3). According to the manufacturer, this treatment produced 2-30µm macroscale roughness. The surface was etched with H2SO4 and HCl, and (again, according to the manufacturer) the microscale roughness obtained was 2-4µm. The surface was then washed with high-purity water. Because of particle size, Rv roughness was lower than in Certain, Interna and Hickelt, and Rp and Rt were lower than in Certain, Interna, SPI and Hikelt. Rp, Rv and Rt were lower, which means that the Surface presented homogenous abrasion (Fig. 1b). The surface was porous due to the HCl and H2SO4 etching that followed blasting (22) 

SPI had similar Ra and Rq scores to Tissue Level and Interna. However, it was the system with the highest Rp score after Certain and Hikelt; this was due to the use of sandblasting, which produced 20-40µm macroscale roughness (according to the manufacturer), and of high temperature double acid etching, which produced 1-5µm microscale roughness. Rt score was intermediate in relation to the other systems, due to this treatment which produces a surface of intermediate porosity, pronounced peaks and marked isolated pores (Fig 1e).

MG-InHex used a RBM (Resorbable Blast Media) treatment consisting of calcium phosphate applied in spray form over a titanium surface. The implant was then passivated, including acid etching. The spray treatment not only produced a rough surface, but also eliminated surface contamination and increased activity on the implant surface (23). The roughness obtained was lower, as the particle size and hardness was lower than those used in the other systems, therefore producing less abrasion than aluminium oxide, carbon or corundum. Because of this, the surface had the lowest Ra and Rq scores. MG-InHex also obtained the lowest Rv, Rp and Rt scores, which showed homogeneity in surface treatment (Fig. 1d).

Skewness (Rsk) measures profile symmetry over midline. This parameter is sensitive to occasional deep valleys or high peaks. Profiles with few peaks and deep valleys have negative Rsk (porous or punctured surface). Profiles with slight valleys and high peaks have positive Rsk (narrow or pointed surface). This parameter distinguishes between surfaces with different profiles but the same Ra or Rq score (11). Certain, Interna and Hikelt had positive Rsk scores; their surfaces were pointed because of blasting. Hikelt only used etching, and the resulting surface had pronounced peaks. Acid etching, without previous abrasion, usually produces negative Rsk; therefore Hickelt surface should not have been pointed. On the contrary, Tissue Level, MG-InHex and SPI had negative Rsk. Their surfaces were porous due to acid etching after blasting. Other studies show that acid etching creates pores on the surface (negative Rsk) (8,15,20). A punctured or porous surface has been proven to be better for cell adhesion, as it has been shown in some studies (20). Likewise it has been proven that cell adhesion is better in a less rough surface with negative Rsk (porous) than in a rougher surface with positive Rsk (15).

Kurtosis (Rku) describes the frequency of the roughness pattern. If Rku is lower than 3, the surface is platykurtic and presents some high peaks and a few valleys. If Rku is higher than 3, the distribution curve is called leptokurtic and presents some high peaks and a few deep valleys (11). It has been shown that a score higher than 3 (leptokurtic surface) has a positive effect on implant retention (20). The score for roughness parameter Rku was lower than 3 in Certain and Hikelt, as a result of being treated with 20-100µm particles in Certain, which results in a very high roughness score, and as a result of an exclusive etching process in Hikelt, which produces pores that are more spaced apart (19). The other four systems had scores higher than 3 and were leptokurtic surfaces.

An ideal roughness score has not been established. A rough surface improves cellular adhesion; however, too rough a surface hinders osseointegration and biological response (24). An ideal Ra roughness range (0.775µm± 0.058µm) and Rt range (5.258µm ± 0.554) have been proposed (15). A maximum Ra score of about 40µm has been established (10). All the systems analysed in this study had lower scores. Eisenbarth *et al.* (25), analysed the effect of surface roughness on cellular adhesion, and found that Ra = 7µm achieved fast cell migration. However, Ra = 40µm achieved less satisfactory cell adhesion. This study also found that a medium Ra (Ra = 15µm) achieved the best cell adhesion, coming to the conclusion that high roughness is not necessary in order to achieve the best cell response. The systems analyzed in this study had lower scores, and were therefore within the recommended roughness range. In any case, we should not take into account only parameter Ra in order to determine the suitability of a surface.

Roughness, topography and morphology patterns varied in each of the implants in this study. Not all implants had the same roughness measurements and topographic characteristics. As we have proven, surface morphology and roughness are directly linked to implant survival, cell adhesion and predictability in treatments where the bone situation is less favorable (26-28). Lastly, surface characterization requires several parameters at the same time in order to reach more certain conclusions.

## Conclusions

Topography and roughness varied in each of the implants analyzed in this study, and it was found that prior acid treatment contributed to negative Rsk and that particle blasting determined positive Rsk.
